# Quality of Life in the First Year of Cancer Diagnosis among Aboriginal and Non-Aboriginal People Living in Regional and Remote Areas of Australia

**DOI:** 10.3390/ijerph19010330

**Published:** 2021-12-29

**Authors:** Elaina Elder-Robinson, Abbey Diaz, Kirsten Howard, Darshit Rajeshkumar Parikh, Giam Kar, Gail Garvey

**Affiliations:** 1 School of Public Health, Faculty of Medicine, The University of Queensland, Herston, QLD 4006, Australia; abbey.diaz@uq.edu.au (A.D.); g.garvey@uq.edu.au (G.G.); 2 Menzies School of Health Research, Charles Darwin University, Casuarina, NT 0810, Australia; 3 Menzies Centre for Health Policy and Economics, School of Public Health, University of Sydney, Sydney, NSW 2006, Australia; kirsten.howard@sydney.edu.au; 4 School of Psychological and Clinical Sciences, Charles Darwin University, Causarina, NT 0810, Australia; darshiitt@gmail.com; 5 Alan Walker Cancer Care Centre, Royal Darwin Hospital, Casuarina, NT 0810, Australia; kar.giam@nt.gov.au

**Keywords:** quality of life, health-related quality of life, AQoL, remote, regional, indigenous health, aboriginal, Northern Territory

## Abstract

Little is known of the quality of life (QoL) of cancer patients in the Northern Territory (NT) of Australia, where healthcare delivery is geographically challenged. This exploratory study describes QoL among Aboriginal and non-Aboriginal cancer patients in the NT, in the first year of diagnosis. Participants were recruited from the only cancer care centre in the NT and completed the Assessment of Quality-of-Life questionnaire (AQoL-4D). The results were descriptively analysed. The participants’ (n = 63; mean age 58.8 years) mean AQoL utility score was 0.72 (SD 0.26); patients scored lowest in the relationships and mental health dimensions of the questionnaire (mean 0.89, SD 0.19, and 0.89, SD 0.17, respectively). Participants living in remote and very remote areas (46%) reported higher QoL scores, compared with participants in the outer regional capital city of the NT in the overall (mean 0.76, SD 0.22 and 0.78, SD 0.20 vs. 0.67, SD 0.29, respectively), and mental health dimensions (mean 0.92, SD 0.09 and mean 0.94, SD 0.06 vs. 0.85, SD 0.22, respectively). The findings were suggestive of clinically meaningful differences across socioeconomic groups, cancer and treatment types, and comorbidity status. Mean QoL scores were consistent with previous reports in other Australian cancer cohorts. The findings suggest a need to support cancer patients’ mental health and relationships during the diagnosis and treatment phase of their cancer journey.

## 1. Introduction

The Northern Territory (NT) is situated in the central and central northern regions of Australia. Despite its large geographical coverage [1] it is sparsely populated, with less than 250,000 residents [2]. Aboriginal and Torres Strait Islander people make up around thirty percent of this population [3], compared with three percent of the total Australian population. The NT is largely classified as very remote (very little access to services) or remote (very restricted access), with only the capital city, Darwin, being considered outer regional (significantly restricted access) [4,5]. Eighty percent of the NT Aboriginal population live in remote or very remote areas. Geographical remoteness presents a challenge for the optimal and equitable delivery of cancer treatment and other health services [6].

Cancer is a leading cause of illness and death in Australia [7]. Compared with other Australian jurisdictions, the Northern Territory had the second lowest age-standardised incidence of all cancers combined, across 2010–2014 (466 cases per 100,000 population), yet the highest age-standardised cancer mortality (212 deaths per 100,000 cases), across 2012–2016 [8]. The NT also had the highest incidences of head and neck, liver, pancreatic and lung cancers, and cancers of an unknown primary site [8]. Based on age-standardised rates, the overall cancer incidence in the Northern Territory was slightly lower for the Aboriginal population, compared with the non-Aboriginal population (431 and 474 per 100,000 population, respectively), although mortality was higher (309 and 200 per 100,000 population, respectively) [9]. National five-year cancer survival rates are improving, although this is to a lesser degree for those living in remote areas [7]. Between 2010–2014, five-year survival was 62 percent in major cities, compared with 55 percent for very remote areas [7].

Cancer diagnosis and treatment can have a profound impact on the quality of peoples’ lives, beyond the commonly measured endpoints of morbidity and mortality [10,11,12,13]. These impacts may be experienced for extended periods of time, and across multiple dimensions of peoples’ lives, including psychological, social, and physical dimensions [13,14]. Assessing and addressing the quality of life (QoL) of cancer patients has been increasingly identified as important, and many survivorship studies and models of survivorship care have included QoL as an important outcome. Identifying and addressing the concerns and needs of cancer survivors is important to optimising their health and wellbeing [13]. However, there is limited information on the QoL of cancer patients in the NT, and limited information on Aboriginal cancer patients, despite the different experiences and epidemiology of cancer in these populations.

An understanding of cancer patients’ QoL can assist tertiary and primary care service providers to respond to the needs of patients, and design and deliver more personalised patient-centred care. In this exploratory study, we describe the QoL of a sample of NT cancer patients and explore variation in QoL associated with sociodemographic and clinical factors.

## 2. Materials and Methods

### 2.1. Study Design and Setting

We utilised a cross-sectional study design to explore factors associated with QoL in cancer patients in the NT, as part of a larger study investigating psychosocial aspects of cancer care. Of the three hospitals in the NT, only one offers comprehensive cancer care, and this site was chosen for the study [6]. This cancer centre treats over 800 cancer patients each year, and offers surgery, chemotherapy, immunotherapy, hormone therapy and radiotherapy.

### 2.2. Participants

The larger study recruited participants between February 2015 and September 2016. Adults (18 years or over) diagnosed with a malignant cancer in the last five years and attending the cancer care centre for further diagnostic investigations, treatment, and follow-up care for their cancer, were invited to participate. As QoL was likely to be dependent on time since diagnosis [13], this analysis was restricted to participants within 12 months of diagnosis. Patients were excluded from the study if they were too ill to participate, or were not able to provide informed consent, as determined by the cancer centre staff. Potentially eligible participants were identified from daily attendance lists and invited to participate by centre staff. Patients who expressed interest were contacted by a member of the research team, who provided further study details and sought written informed consent before participating in an interview.

### 2.3. Data Collection and Measurement Tool

Face-to-face interviews were conducted by a trained member of the research team (DP) at a time and place convenient to both the patient and the interviewer. QoL was assessed during the interview using the Assessment Quality-of-Life questionnaire (AQoL-4D) [15,16]. The AQoL-4D is a multi-attribute measure of QoL, with scoring algorithms that combine responses into dimension scores, and a single overall score. The AQoL-4D has been previously used by members of our team among a cohort of Aboriginal cancer patients in Queensland, Australia [10]. The AQoL-4D contains four dimensions, each with three items: independent living (self-care, household tasks, and mobility); relationships (friendships, isolation and family role); sense (seeing, hearing and communication); and mental health (sleeping, worrying and pain). Each item has four response levels to indicate the degree to which the QoL within a specific domain was impacted. It can be scored as either a simple additive psychometric score, or as a preference-based utility score for overall and dimension-specific values.

The interview also captured self-reported socio-demographic data, including age, gender, Indigenous status, marital status, employment status, educational attainment, main language spoken at home, and residence by postcode and community name [17]. Medical chart reviews were conducted using a standard data collection form by a member of the cancer care team to gather participant’s clinical information (diagnosis date, cancer staging at diagnosis, cancer type, and treatment regimen (current or planned treatments).

Outcome measure: responses to the 12 QoL questions were used to calculate overall AQoL utility scores for participants using standard scoring algorithms obtained from the Assessment of Quality-of-Life website [15]. Dimension utility scores for independent living, relationships, sense, and mental health, were also calculated using the published algorithms. Utility scores are preference scores that lie on a scale of 0.00 (death) to 1.00 (full health). The AQoL-4D can take scores ranging from −0.04 to 1.0, and therefore includes the valuation of health states worse than death. Population norms suggest an overall utility score greater than 0.90 represents excellent QoL [18], and a difference of around 0.06 may be clinically meaningful [19]. There were no missing data for the 12 QoL questions. We use the term ‘utility score’ throughout to refer to the QoL utility scores generated from the AQoL-4D measure.

Exploratory factors: where cell count size was less than five, categories of clinical and socio-demographic variables were collapsed, or not reported, to avoid potential re-identification of participants [20]. Therefore, most characteristics were reported as dichotomous groupings: age at interview (above and below the median age); gender (male and female); Indigenous status (Aboriginal and non-Aboriginal); marriage status (partnered and not partnered); educational attainment (Year 10 or below and above Year 10); employment status (employed and not employed); language spoken at home (English and not English); comorbidities (none, one comorbidity, between two and five comorbidities); cancer type (breast cancers, cancers of the head and neck, cancers of the digestive system, skin cancers and other cancers); treatment modality (surgery with or without radiotherapy, chemotherapy and hormone therapy; treatment excluding surgery); cancer stage at diagnosis (local/regional, advanced, unknown/not applicable); and time since diagnosis (equal or less than three months (median), or between three and twelve months).

Participants postcodes and community names were mapped to the 2006 Australian Remoteness Index of Areas (ARIA) [5], and participants were classified as living in ‘Outer Regional’ (significantly restricted accessibility), ‘Remote’ (very restricted accessibility) and ‘Very Remote’ (very little accessibility) areas, due to there being no inner regional or major city areas in the NT, which are characterised by greater accessibility to goods, services and social opportunities [4,5]. This classification reflects the participants’ home location and does not encompass time spent away from their home whilst receiving cancer treatment. Postcodes were also mapped to the Index of Socio-economic Advantage and Disadvantage (IRSAD) and due to small cell size, categorised as least advantaged (deciles 1–5) and most advantaged (deciles 6–10) [21].

### 2.4. Data Analysis

The participants’ sociodemographic and clinical characteristics were described using frequencies and proportions. Mean and standard deviation for overall and dimension-specific utility scores were reported. Simple linear regression models were conducted to explore crude associations between AQoL-4D utility scores and socio-demographic and clinical characteristics of the participants. Dimension-specific utility scores were presented, stratified across sub-groups. Statistical significance tests were not conducted due to the exploratory nature of the study and small sample sizes. All analysis was conducted in Stata, version 15 [22].

### 2.5. Ethics

Ethics approval for this study was obtained from the Northern Territory Health Department and the Menzies School of Health Research Human Research Ethics Committee (#13-2038).

## 3. Results

In total, 225 potentially eligible patients were identified. Not all could be approached by the centre staff for recruitment, due to staff time constraints or patient absence from appointment. In total, 89 patients were approached and invited to participate (39% of those eligible). Of these patients, 76 (85% of invited) consented, and completed the interview. All Aboriginal patients (n = 30) who accessed the centre were invited, and 87% consented and participated in the study.

In total, 63 NT cancer patients who were within the first 12 months of their cancer diagnosis (median 3 months since diagnosis) were included in the analysis. Participant characteristics are described in Table 1. Participants were aged 58.8 years (mean, SD 13.7) at the time of interview, 49% were women and 32% were Aboriginal. Almost half of the participants lived in remote or very remote areas (46%) and almost one-third lived in the most socioeconomically disadvantaged areas (35%). The two most common cancers were breast (29%) and head and neck (14%) cancers.

**Figure 1:**
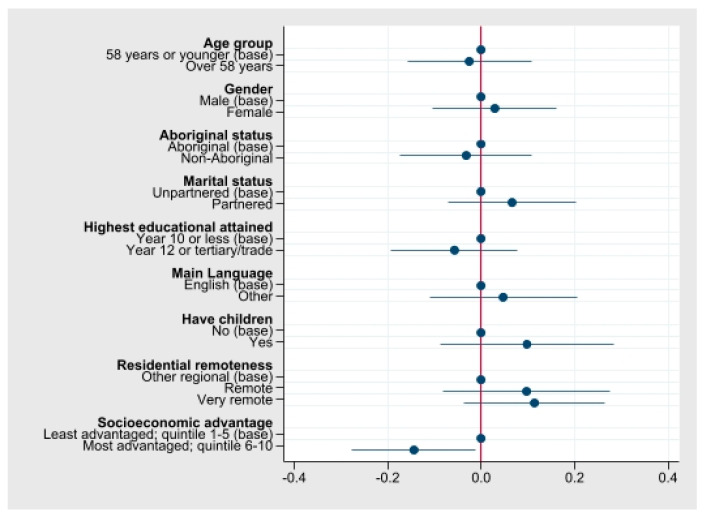
Crude associations between AQoL-4D utility scores and participant socio-demographic characteristics in cancer survivors (n = 63) in the first year of diagnosis, NT, Australia.

The mean overall utility score was 0.72 (SD 0.26). Associations between AQoL-4D utility scores and socio-demographic characteristics are shown in Figure 1, with underlying stratified mean scores presented in Supplementary Table S1. The crude regression models suggest remoteness, socioeconomic advantage, having children, and having a partner, may have been associated with AQol-4D utility scores (Figure 1). Moreover, scores may be highest among cancer patients living in remote and very remote areas, compared with the outer regional capital city areas of the NT (mean scores 0.76, 0.78 and 0.67 respectively; Supplementary Table S1). Participants in the least advantaged areas had a higher mean utility score, compared with those in the most advantaged areas (mean scores 0.81 and 0.67). Participants without children had a higher utility score compared with those with children (mean scores 0.80 and 0.70), and those living with a partner had a higher utility score, compared with those living without a partner (mean scores 0.74 and 0.67). The data suggests mean utility scores were similar across gender, age group, and Indigenous status.

**Figure 2:**
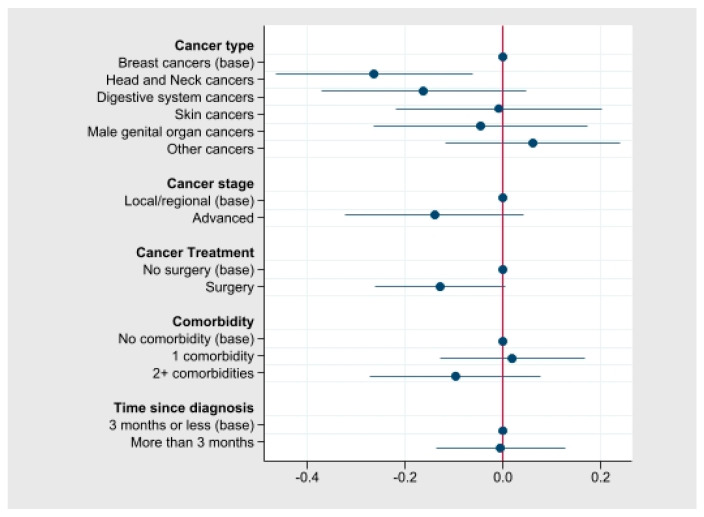
Crude associations between AQoL-4D utility scores and participant clinical characteristics in cancer survivors (n = 63) in the first year of diagnosis, NT, Australia.

Associations between AQoL-4D utility scores and clinical characteristics of the participants are described in Figure 2, with the underlying mean utility scores in Supplementary Table S1. Mean utilities scores were the highest among participants with breast cancers or skin cancers (mean scores 0.77 and 0.76, respectively; Supplementary Table S1) compared with those with digestive system cancers, and head and neck cancers (mean scores 0.60 and 0.50). Participants who had completed surgical treatment, with or without receiving another form of treatment (radiotherapy, chemotherapy, hormone therapy), reported a higher utility score than those were receiving or had completed treatment types other than surgery (mean scores 0.77 and 0.64).

Table 1 reports utility scores for the four dimensions: independent living, relationships, senses and mental health. The scores for independent living (mean 0.92, SD 0.15) and the senses dimensions (mean 0.96, SD 0.09) were high among the participants. The relationship (mean 0.89, SD 0.19) and mental health dimensions (mean 0.89, SD 0.17) utility scores were lower, with greater variability between sub-groups. The data suggest mean utility scores may be lower in the relationship dimension for those with children (mean, 0.83, SD 0.18) than those without children (mean 0.90, SD 0.25), and for head and neck cancer patients (mean 0.74, SD 0.30) compared with those with other cancer types. For the mental health dimension, mean utility scores may be lower for: Aboriginal patients (mean 0.86, SD 0.05) than non-Aboriginal patients (mean 0.94, SD 0.20); outer regional patients (mean 0.85, SD 0.22) than patients from remote (mean 0.92, SD 0.09) and very remote locations (mean 0.94, SD 0.06); and patients from the most socioeconomically advantaged locations (mean 0.86 SD 0.20) compared with the least advantaged locations (mean 0.94, SD 0.06).

## 4. Discussion

Overall, results of this exploratory study indicated a high QoL among NT cancer patients in the first year of diagnosis. The mean utility score for study participants (0.72) was lower than that reported for the general Australian population (0.81) but was similar to that reported for other Australian cancer populations (0.74) [18].

Average utility scores were similar for Aboriginal and non-Aboriginal participants in the current study, except in the mental health domain. Although the scores for Aboriginal participants were higher than the mean score reported previously for Queensland Aboriginal and Torres Strait Islander cancer patients in the first six months of diagnosis (0.62) [10,23], the finding of similar QoL scores between Indigenous and non-Indigenous cancer patients has also been reported in the United States [24,25].

A recent study on unmet supportive care needs found that more than one in three NT cancer patients had unmet needs related to physical and psychological concerns and practical and cultural concerns, with worries about money and finding accommodation during treatment the two most frequently experienced unmet needs [14]. Aboriginal cancer patients are more likely to live outside of the capital city than non-Aboriginal cancer patients. In the current study, however, participants who lived in remote and very remote areas had higher QoL scores than those who lived in outer regional areas; a finding that was also reported in the previous Queensland study [10].

Similar to the pattern observed for remoteness, participants in the current study from socioeconomically disadvantaged areas had higher mean QoL scores than those from more affluent areas, particularly in the mental health dimension. These findings may appear counter-intuitive, given cancer patients in these areas face additional barriers to healthcare access, including accessing cancer treatments, associated travel and accommodation difficulties, and financial burdens [26,27]. Indeed previous work using a population of Aboriginal and non-Aboriginal NT cancer patients published by our team, that overlaps with the current study sample, suggests variable access to community and health services based on demographic variables [28], with barriers to accessing cancer treatment services including complicated and delayed treatment commencement, and challenges related to transport and accommodation [29]. The higher QoL scores reported here, regardless of these challenges, may reflect the presence of other drivers of QoL, such as connections to land, language, and culture. Further research is needed to better understand how to support QoL in cancer patients across levels of remoteness and socioeconomic advantage.

Participants living with a partner reported higher utility scores in the current study, similar to what has been reported in previous cancer populations [10] and Australian population norms [18]. We also found some differences in mean utility according to whether the participant had children. Lower scores were reported for the relationship dimension, yet higher scores were reported for the independent living and mental health dimensions for participants who had children. This may be attributed to higher levels of worry experienced by people with cancer when they have partners, friends and/or family in their lives [30], or persons caring for them during the treatment process [14], as has been reported in previous studies by Australian [30] and Aboriginal and Torres Strait Islander people with cancer [14]. A better understanding of how social relationships, which may be indicative of both support and responsibilities, may affect QoL in cancer patients is warranted.

QoL varies by cancer type, due to the heterogenous impacts of varying treatment protocols on functional status and everyday life [13], and this was reflected in the utility scores reported in the current study. Breast cancer may be associated with higher utility scores, whilst head and neck cancers and digestive system cancers may be associated with the lowest utility scores. Digestive cancers and head and neck cancers particularly seemed to impact the relationship dimension, and head and neck cancers may also be associated with lower utility scores in the mental health dimension. These differences may reflect the more severe and longer-term side effects that head and neck cancer patients can face, such as difficulties with speech, swallowing, and eating, which impact daily life and have significant psychosocial effects [31].

The current study aimed to explore potential drivers of QoL for Aboriginal and non-Aboriginal cancer patients living in the geographically dispersed Northern Territory of Australia, to inform the development of larger, statistically powered studies. Other studies have reported on the psychosocial aspects of cancer care for cancer patients in the Northern Territory [14,28,29]; however, this is the first study to report on the QoL of this specific patient population. A limitation of this study design that needs to be considered includes the fact that only patients who were attending a single cancer service were invited to participate in this study. As such, it is likely that these findings underestimate the QoL of NT cancer patients not able to access this service, or who accessed other cancer services. We make two key recommendations for future studies. First, based on our findings, we recommend future studies are designed to enable the investigation of drivers of QoL, and the piloting of potential strategies to improve QoL separately for patients living in remote, regional and urban areas, as the challenges patients face after their cancer diagnosis and during their cancer treatment are likely to be vastly different based on their place of residence. Second, our analysis used data from a small sample of participants within the first year of their cancer diagnosis. However, given there is so much change for a person in the first year of their diagnosis, and QoL tends to be lower in the early stages of cancer diagnosis and treatment [13], there is likely to have been variation in QoL based on the time since diagnosis. We recommend sub-group analyses that can explore drivers of QoL at specific time points along the cancer continuum.

We further recommend that future studies involving Aboriginal and Torres Strait Islander people, consider the validity of using common tools to measure QoL, and other psychosocial measures, for this population group. An increasing body of work is focusing on the identification of concepts of health and wellbeing from Indigenous perspectives [24,32,33,34,35]; however, currently available QoL measures have been developed through a Western-centric lens and may not include all relevant concepts for Indigenous Australians [33]. Members of our research team are currently leading the development of a new preference-based and holistic measure of wellbeing for Aboriginal and Torres Strait Islander adults, through methodology that privileges the voices of Aboriginal and Torres Strait Islander people and communities [33,35]. A future adaptation of this tool for Aboriginal and Torres Strait Islander people with cancer may be a more valid measure of what matters to Aboriginal and Torres Strait Islander cancer patients, than existing health-related QoL measures.

## 5. Conclusions

This study explored variations in QoL across socio-demographic and clinical factors for Aboriginal and non-Aboriginal NT cancer patients in the first year of diagnosis. Although QoL was generally high, there were notable variations in QoL across cancer types and places of residence that warrant further investigation. Such evidence is needed to inform the development of interventions that aim to improve the quality of survivorship for cancer patients living in the NT.

## Figures and Tables

**Table 1:** AQoL-4D dimension scores by characteristics in cancer survivors in their first year of diagnosis, NT, Australia.

	N (%)	Independent Living	Relationships	Senses	Mental Health
		Mean (SD)	Mean (SD)	Mean (SD)	Mean (SD)
Total participants	63 (100)	0.92 (0.15)	0.89 (0.19)	0.96 (0.09)	0.89 (0.17)
*Age group* (mean 58.8, SD 13.70)					
≤58 years	32 (51)	0.92 (0.15)	0.87 (0.22)	0.98 (0.05)	0.89 (0.17)
>58 years	31 (49)	0.91 (0.14)	0.90 (0.16)	0.94 (0.12)	0.88 (0.18)
*Gender*					
Male	32 (51)	0.91 (0.15)	0.89 (0.19)	0.93 (0.12)	0.89 (0.17)
Female	31 (49)	0.92 (0.15)	0.89 (0.19)	0.98 (0.05)	0.88 (0.18)
*Indigenous Status*					
Aboriginal	20 (32)	0.90 (0.12)	0.87 (0.26)	0.97 (0.04)	0.86 (0.05)
Non-Aboriginal	43 (68)	0.92 (0.16)	0.90 (0.15)	0.95 (0.11)	0.94 (0.20)
*Marital Status*					
Partnered	40 (63)	0.90 (0.17)	0.94 (0.09)	0.96 (0.09)	0.88 (0.20)
Unpartnered	23 (37)	0.94 (0.10)	0.81 (0.27)	0.95 (0.11)	0.89 (0.11)
*Highest level of educational attainment*					
Year 10 education	39 (62)	0.90 (0.17)	0.91 (0.18)	0.97 (0.08)	0.90 (0.19)
Year 12, trade, or tertiary	24 (38)	0.93 (0.10)	0.86 (0.20)	0.94 (0.11)	0.87 (0.14)
*Main language spoken at home*					
English	49 (78)	0.92 (0.16)	0.89 (0.17)	0.95 (0.11)	0.87 (0.19)
Other	14 (22)	0.90 (0.12)	0.89 (0.25)	0.97 (0.04)	0.94 (0.05)
*Children*					
No Children	9 (14)	0.99 (0.04)	0.90 (0.25)	0.95 (0.05)	0.87 (0.05)
Children	54 (86)	0.90 (0.16)	0.83 (0.18)	0.98 (0.10)	0.96 (0.18)
*Remoteness Category*					
Outer Regional	34 (54)	0.90 (0.17)	0.88 (0.16)	0.95 (0.12)	0.85 (0.22)
Remote	11 (17)	0.95 (0.11)	0.89 (0.23)	0.97 (0.05)	0.92 (0.09)
Very Remote	18 (29)	0.92 (0.11)	0.91 (0.22)	0.96 (0.07)	0.94 (0.06)
*Socioeconomic advantage ^a^*					
Least advantaged	22 (35)	0.94 (0.10)	0.93 (0.20)	0.96 (0.06)	0.94 (0.06)
Most advantaged	41 (65)	0.90 (0.17)	0.87 (0.18)	0.95 (0.11)	0.86 (0.20)
*Comorbidities*					
0	31 (49)	0.93 (0.15)	0.85 (0.23)	0.97 (0.05)	0.91 (0.09)
1	23 (37)	0.91 (0.12)	0.95 (0.08)	0.97 (0.07)	0.87 (0.22)
2–5	9 (14)	0.88 (0.18)	0.87 (0.21)	0.90 (0.17)	0.87 (0.22)
*Cancer Type*					
Breast	18 (29)	0.91 (0.18)	0.87 (0.22)	0.99 (0.03)	0.94 (0.08)
Head and neck	9 (14)	0.92 (0.12)	0.74 (0.30)	0.90 (0.15)	0.81 (0.30)
Digestive organs	8 (12)	0.85 (0.24)	0.86 (0.12)	0.90 (0.18)	0.90 (0.06)
Skin	8 (12)	0.90 (0.11)	0.95 (0.14)	0.94 (0.08)	0.89 (0.15)
Male Genital Organ	7 (11)	0.93 (0.12)	0.93 (0.10)	0.99 (0.03)	0.79 (0.30)
Other ^b,c^	13 (21)	0.95 (0.09)	0.97 (0.07)	0.98 (0.03)	0.91 (0.19)
*Stage*					
Local/Regional	28 (44)	0.95 (0.10)	0.91 (0.19)	0.97 (0.09)	0.91 (0.18)
Advanced	18 (29)	0.93 (0.22)	0.87 (0.19)	0.96 (0.06)	0.82 (0.21)
Unknown	17 (27)	0.94 (0.08)	0.87 (0.19)	0.94 (0.12)	0.91 (0.08)
*Treatment Type*					
Surgery +/− other treatment	35 (56)	0.93 (0.14)	0.90 (0.15)	0.97 (0.08)	0.91 (0.16)
Treatment without surgery ^d^	26 (41)	0.89 (0.16)	0.86 (0.23)	0.93 (0.11)	0.85 (0.19)
*Time Since Diagnosis*					
≤3 months	32 (51)	0.95 (0.08)	0.85 (0.24)	0.94 (0.10)	0.91 (0.10)
3–12 months	31 (49)	0.87 (0.19)	0.93 (0.09)	0.97 (0.09)	0.86 (0.22)

Abbreviations: AQoL-4D: Australian Quality-of-Life–4 Dimension Index; NT: Northern Territory; SD: standard deviation. Notes: ^a^. Socioeconomic Index for Areas (SEIFA) index dichotomized as least disadvantaged (deciles 1–5) and most advantaged (quintiles 6–10); ^b^. Other cancer types included: eye, brain and central nervous system cancers; ill-defined, secondary and unspecified cancers; female genital organ cancers; respiratory and intrathoracic organs; lymphoid, haematopoietic and related tissue cancers; ^c^. Missing data n = 2; ^d^. Other treatment types include chemotherapy, radiotherapy and hormone therapy.

## Data Availability

The data presented in this study are not available due to ethical conditions and consent agreements, Queries about analyses can be directed to the corresponding author.

## Supplementary Materials

The following supporting information can be downloaded at: https://www.mdpi.com/article/10.3390/ijerph19010330/s1, Table S1: AQoL-4D overall mean (SD) utility scores by characteristics in cancer survivors in their first year of diagnosis, NT, Australia.
