# Supplementary material for: Quality of Life in the First Year of Cancer Diagnosis among Aboriginal and Non-Aboriginal People Living in Regional and Remote Areas of Australia
Source: Int J Environ Res Public Health. 2021 Dec 29;19(1):330. doi: 10.3390/ijerph19010330 (PMC8751055; doi:10.3390/ijerph19010330)
Supplement: Supplementary file 1 [file ijerph-19-00330-s001.zip › ijerph-1509862-supplementary.pdf]

Supplementary Table S1

AQoL-4D overall utility scores by characteristics in cancer survivors in their first year of diagnosis, NT, Australia.

	N (%)	AQoL Overall utility Score	
		Mean (SD)	Median (IQR)
Overall	63 (100)	0.72 (0.26)	0.74 (0.62, 0.95)
Age			
Mean (SD); median (IQR)	58.8 (13.70); 58 (48, 69)		
≤58 years	32 (51)	0.73 (0.27)	0.77 (0.65, 0.95)
>58 years	31 (49)	0.70 (0.26)	0.73 (0.57, 0.95)
Gender			
Male	32 (51)	0.70 (0.26)	0.74 (0.62, 0.92)
Female	31 (49)	0.73 (0.26)	0.75 (0.62, 0.98)
Indigenous Status			
Aboriginal	20 (32)	0.74 (0.22)	0.76 (0.66, 0.86)
Non-Aboriginal	43 (68)	0.71 (0.28)	0.74 (0.56, 0.98)
Marital Status			
Partnered (married/de facto)	40 (63)	0.74 (0.25)	0.76 (0.65, 1.00)
Unpartnered (single, divorced, widowed)	23 (37)	0.67 (0.27)	0.72 (0.47, 0.95)
Highest level of educational attainment			
Year 10 education	39 (62)	0.74 (0.27)	0.81 (0.63, 0.98)
Year 12, trade, certificate/ diploma, university	24 (38)	0.68 (0.24)	0.72 (0.56, 0.84)
Main language spoken at home			
English	49 (78)	0.70 (0.27)	0.73 (0.57, 0.98)
Other	14 (22)	0.75 (0.21)	0.78 (0.72, 0.86)
Children			
No Children	9 (14)	0.80 (0.25)	0.92 (0.72, 1.00)
Children	54 (86)	0.70 (0.26)	0.74 (0.61, 0.92)
Remoteness Category			
Outer Regional	34 (54)	0.67 (0.29)	0.72 (0.47, 0.98)
Remote	11 (17)	0.76 (0.22)	0.81 (0.63, 0.98)
Very Remote	18 (29)	0.78 (0.20)	0.79 (0.71, 0.95)
SEIFA score (deciles)[a]			
1-5	22 (35)	0.81 (0.20)	0.81 (0.74, 0.98)
6-10	41 (65)	0.67 (0.28)	0.73

	N (%)	AQoL Overall utility Score	
		Mean (SD)	Median (IQR)
			(0.48, 0.92)
Comorbidities			
0	31 (49)	0.73 (0.25)	0.78 (0.63, 0.93)
1	23 (37)	0.75 (0.25)	0.74 (0.63, 0.98)
2-5	9 (14)	0.63 (0.31)	0.73 (0.36, 0.85)
Cancer Type			
Breast	18 (29)	0.77 (0.24)	0.83 (0.63, 0.98)
Head and neck	9 (14)	0.50 (0.32)	0.48 (0.25, 0.72)
Digestive organs	8 (12)	0.60 (0.19)	0.64 (0.48, 0.72)
Skin	8 (12)	0.76 (0.27)	0.79 (0.60, 1.00)
Male Genital Organ	7 (11)	0.72 (0.32)	0.73 (0.67, 0.98)
Other[b, c]	13 (21)	0.83 (0.22)	0.81 (0.74, 0.98)
Stage			
Local/Regional	28 (44)	0.79 (0.27)	0.93 (0.69, 1.00)
Advanced	18 (29)	0.60 (0.26)	0.65 (0.36, 0.78)
Unknown	17 (27)	0.71 (0.19)	0.74 (0.66, 0.84)
Treatment Type			
Surgery with/without other treatment	35 (56)	0.77 (0.25)	0.84 (0.64, 1.00)
Treatment without surgery[d]	26 (41)	0.64 (0.27)	0.73 (0.47, 0.81)
Time Since Diagnosis			
≤3 months	32 (51)	0.72 (0.24)	0.75 (0.62, 0.93)
3-12 months	31 (49)	0.71 (0.28)	0.74 (0.62, 1.00)

Abbreviations: AQoL-4D: Australian Quality of Life – 4 Dimension Index; NT: Northern Territory; SD: Standard deviation

NOTES: a. Socioeconomic Index for Areas (SEIFA) index dichotomized as least disadvantaged (deciles 1-5) and most advantaged (quintiles 6-10); b. Other cancer types included: eye, brain and central nervous system cancers; ill-defined, secondary and unspecified cancers; female genital organ cancers; respiratory and intrathoracic organs; lymphoid, haematopoietic and related tissue cancers; c. Missing data n=2; d. Other treatment types include chemotherapy, radiotherapy and hormone therapy.
